# The impact of high nicotine concentrations on the viability and cardiac differentiation of mesenchymal stromal cells: a barrier to regenerative therapy for smokers

**DOI:** 10.3389/fcell.2024.1323691

**Published:** 2024-04-04

**Authors:** Maryam Gheisari, Shadi Nosrati, Shahrokh Zare, Mahintaj Dara, Samaneh Zolghadri, Iman Razeghian-Jahromi

**Affiliations:** ^1^ Department of Biochemistry, Shiraz Branch, Islamic Azad University, Shiraz, Iran; ^2^ Stem Cells Technology Research Center, Shiraz University of Medical Sciences, Shiraz, Iran; ^3^ Department of Biology, Jahrom Branch, Islamic Azad University, Jahrom, Iran; ^4^ Cardiovascular Research Center, Shiraz University of Medical Sciences, Shiraz, Iran

**Keywords:** nicotine, mesenchymal stromal cells, viability, cardiac differentiation, cardiovascular risk factors

## Abstract

**Background:** Current treatment methods are not successful in restoring the lost cardiomyocytes after injury. Stem cell-based strategies have attracted much attention in this regard. Smoking, as a strong cardiovascular risk factor, not only affects the cardiac cells adversely but also deteriorates the function of stem cells. Since mesenchymal stem cells (MSCs) are one of the popular candidates in cardiovascular disease (CVD) clinical trials, we investigated the impact of nicotine on the regenerative properties (viability and cardiac differentiation) of these cells.

**Methods:** MSCs were isolated from rat bone marrow and characterized based on morphology, differentiation capability, and the expression of specific mesenchymal markers. The MTT assay was used to assess the viability of MSCs after being exposed to different concentrations of nicotine. Based on MTT findings and according to the concentration of nicotine in smokers’ blood, the growth curve and population doubling time were investigated for eight consecutive days. Cells were treated with 5-azacytidine (an inducer of cardiac differentiation), and then the expressions of cardiac-specific markers were calculated by qPCR.

**Results:** MSCs were spindle-shaped, capable of differentiating into adipocyte and osteocyte, and expressed CD73 and CD90. The viability of MSCs was reduced upon exposure to nicotine in a concentration- and time-dependent manner. The growth curve showed that nicotine reduced the proliferation of MSCs, and treated cells needed more time to double. In addition, the expressions of GATA4 and troponin were downregulated in nicotine-treated cells on day 3. However, these two cardiac markers were overexpressed on day 7.

**Conclusion:** Nicotine decreased normal growth and reduced the expression of cardiac markers in MSCs. This aspect is of eminent importance to smokers with cardiovascular disease who are candidates for stem cell therapy.

## Introduction

A major contributor to the global mortality, disability, and disease burden is cardiovascular disease (CVD), principally ischemic heart disease and stroke. The growing trend of cardiovascular patients, which has doubled from 1990 to 2019, necessitates urgent action for designing and implementing more efficient therapeutic strategies (Roth et al., 2020). New policies resort to using the potential of stem cells, and thereby, nearly one-fifth of all stem cell-based clinical trials in the new decade of 2020 were designed to be conducted in the area of CVD (Chan and Huang, 2020). Despite promising findings, the clinical translation of stem cell-based therapeutics has not reached a consensus yet. One of the important barriers is that the benefits of stem cells are influenced by the health conditions of cell donors and recipients. Good manufacturing practice suggests screening both sides against comorbidities prior to transplantation (Sensebé et al., 2013).

An overlooked aspect of failure in stem cell therapy is smoking status, which seems to considerably affect the final output. Tobacco is the most prevalent risk factor contributing to fatal events in the world (Onor et al., 2017). Other than the detrimental systemic effects caused by nicotine and its metabolites, they specifically impair the function of the cardiovascular system. Smoking increases the risk of atherosclerosis and angiogenesis progression (Santanam et al., 2012; Fu et al., 2021). In addition, nicotine decreases blood flow to the coronary arteries, leading to ischemia (Whitehead et al., 2021), and upon repetitive incidents of acute ischemia, myocardial infarction would be inevitable (Lee and Cooke, 2011). Although the deleterious effects of nicotine on the components of the cardiovascular system have well been documented, little is known about the effects of smoking/nicotine on stem cells.

One of the popular stem cell types used in cardiovascular clinical studies is mesenchymal stromal cells (MSCs) (Chamberlain et al., 2007; Madrigal et al., 2014). It is of eminent importance to evaluate the effects of nicotine on the efficiency of stem cell therapy. The majority of clinical trials include smokers donating or receiving MSC therapy, who may face unknown suboptimal therapeutic efficiency (Greenberg et al., 2017). So, we investigated the viability and cardiac differentiation of MSCs after being exposed to a nicotine concentration near that of smokers’ blood.

## Methods

### MSC extraction and culture

All experiments in this case-control study were performed in accordance with relevant guidelines and regulations and were approved by the regional ethics committee. The study is reported in line with the ARRIVE guidelines. Male adult rats (weight = 110–140 g) were obtained from a valid local animal house. To have a homogenous stem cell pool, three rats were used for the extraction of MSCs. In brief, euthanization was induced by an IV injection of ketamine combined with xylazine (<200 µlit). After the dissection of femurs and tibias, bone extremities were flushed with DMEM enriched with penicillin/streptomycin in order to collect cells within the bone marrow. The obtained cell suspension was subjected to centrifugation. The cell pellet was dissolved in DMEM/FBS and added to a T25 culture flask prefilled with a complete culture medium. Following incubation in an incubator (humidified atmosphere, 5% CO_2_, 37°C), the surface of the flask became confluent after approximately 2 weeks. At this point, cells were detached by trypsin/EDTA treatment and underwent repeated subcultures until reaching passage three (P3). In this study, all the experiments were performed on MSCs at P3.

### Characterization of proliferated cells at P3

In order to be assured about the identity of proliferated cells at P3, they should be characterized based on three criteria: morphology, multilineage differentiation ability, and expression of mesenchymal markers. Morphology was evaluated by inverted microscopy. The ability of the cells to differentiate was checked by culturing cells in adipogenic and osteogenic induction media. In 6-well plates, MSCs were cultured and allowed to expand. When nearly 80% of the well surface was covered by the cells, the culture medium was replaced by adipogenic medium, including Ham’s F-12, 15% FBS, 0.2 mM L-glutamine, 100 μM L-ascorbic acid (Merck), 200 μM indomethacin, and 100 nM dexamethasone. After approximately 21 days, cells were washed and stained with Oil Red O dye. For osteogenic differentiation, cells in 6-well plates were cultured in a specific medium for osteogenesis containing low-glucose DMEM supplement with 0.05 mM ascorbate-2-phosphate (Wako Chemicals, Richmond, VA, United States), 10 mM b-glycerophosphate, 100 nM dexamethasone, 10% FBS, and 1% antibiotic/antimycotic. Cells were washed 21 days later and then stained with alizarin red dye. For the expression of mesenchymal-specific markers, a confluent T75 culture flask was prepared and sent for flow cytometry analyses with CD45 and CD90.

### Viability of nicotine-treated MSCs by the MTT assay

MSCs were prepared on three 24-well plates. After an incubation period of 24 h, different concentrations of nicotine, from 1 nM to 10*10^6^ nM, were added to each well. The plate was kept in the incubator for 1, 2, or 3 days. On the corresponding days, the culture medium was replaced with an MTT stain. After 4 hours, the plate was centrifuged, and the medium was substituted with DMSO. Finally, absorbance was recorded at a wavelength of 570 nm using a spectrophotometer. At the end of this step, a nicotine concentration was selected for the following experiments, which was determined based on the findings of the MTT assay and nicotine concentration in the smokers’ blood (Fagerström et al., 2002). All the experiments were performed three times.

### Growth curve and population doubling time of MSCs treated with nicotine

MSCs were cultured on two 24-well plates. Each well contained 37,500 cells/mL. One plate was considered for the case group whose cells were exposed to the routine culture medium and 100 nM nicotine, and the other plate was MSCs of the control group that were exposed to the culture medium and PBS. After 24 h, three wells were considered in each plate. Their culture medium was removed, and cells were washed with PBS and treated with trypsin/EDTA. After centrifugation, the cell pellet was re-suspended in the culture medium, and a certain volume of the cell suspension was mixed with the same volume of trypan blue stain. This mixture was loaded into a Neubauer chamber. The number of cells in each well was calculated. The mean number of cells in three wells was considered the number of cells on the first day. This procedure was repeated for a further seven consecutive days. All the experiments were performed three times.

### Assessment of morphology and expression of cardiac-specific markers in MSCs treated with nicotine

As the expression of cardiac genes in the case and control groups was intended to be quantified in 2 days (days 3 and 7), MSCs were cultured in two pairs of T25 culture flasks. MSCs were first exposed to 5-azacytidine for 24 h. After this period of cardiogenic induction, cells were treated with either 100 nM nicotine or the same volume of PBS as the case and control groups. On days 3 and 7, the appearance of cells in both the case and control groups was monitored by inverted microscopy. On the corresponding days, the culture medium was removed, and cells were washed with PBS and treated with trypsin/EDTA. Following centrifugation of the suspension, the cell pellet was yielded and subjected to RNA extraction (Rnx Plus Solution, CinnaGen Co). The quality and quantity of RNA were examined by NanoDrop. Using the reverse transcriptase enzyme, cDNA was synthesized (AddScript cDNA Synthesis Kit, addbio), and then qPCR (RealQ Plus 2x Master Mix Green, Ampliqon) was performed using cardiac-specific primers of GATA4 and troponin (Table 1). This experiment was repeated two times. Differential gene expression was revealed between the case and control groups on days 3 and 7. All the experiments were performed three times.

**TABLE 1:** Primer sequences of cardiac markers used in qPCR.

	Forward primer	Reverse primer
GATA4	TGATGGATGGAAGAAGAT	GTGATGAAGACAAGGAAG
Troponin	CAGAGTATCCACAACCTA	CAG​TTC​CAT​CTA​TTT​CCA​A
GAPDH	AAA​CCC​ATC​ACC​ATC​TTC​CA	CAC​GAC​ATA​CTC​AGC​ACC​A

### Statistical analysis

The Statistical Package for Social Sciences (SPSS, V16) was used for analysis. The case and control groups were compared by either an independent sample t-test or ANOVA. Differential gene expression was assessed by the 2^^−ΔΔCt^ method. A *p*-value of <0.05 was considered statistically significant.

## Results

### Characterization of the proliferated cells at P3

**FIGURE 1:**
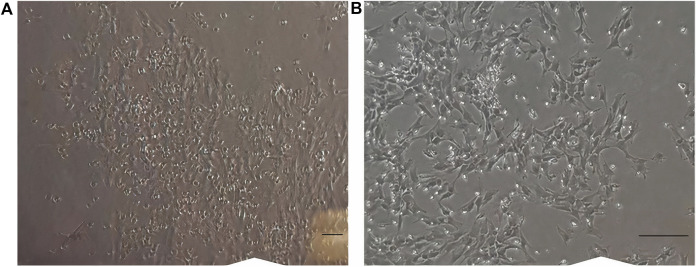
**(A)** Cells in earlier cultures. Different appearances, like flattened, spindle, and multi-dimensional, were seen. **(B)** Cells at passage 3. Most of the cells are spindle-shaped or fibroblast-like. Scale bar shows 100 μm (magnification is 360x).

**FIGURE 2:**
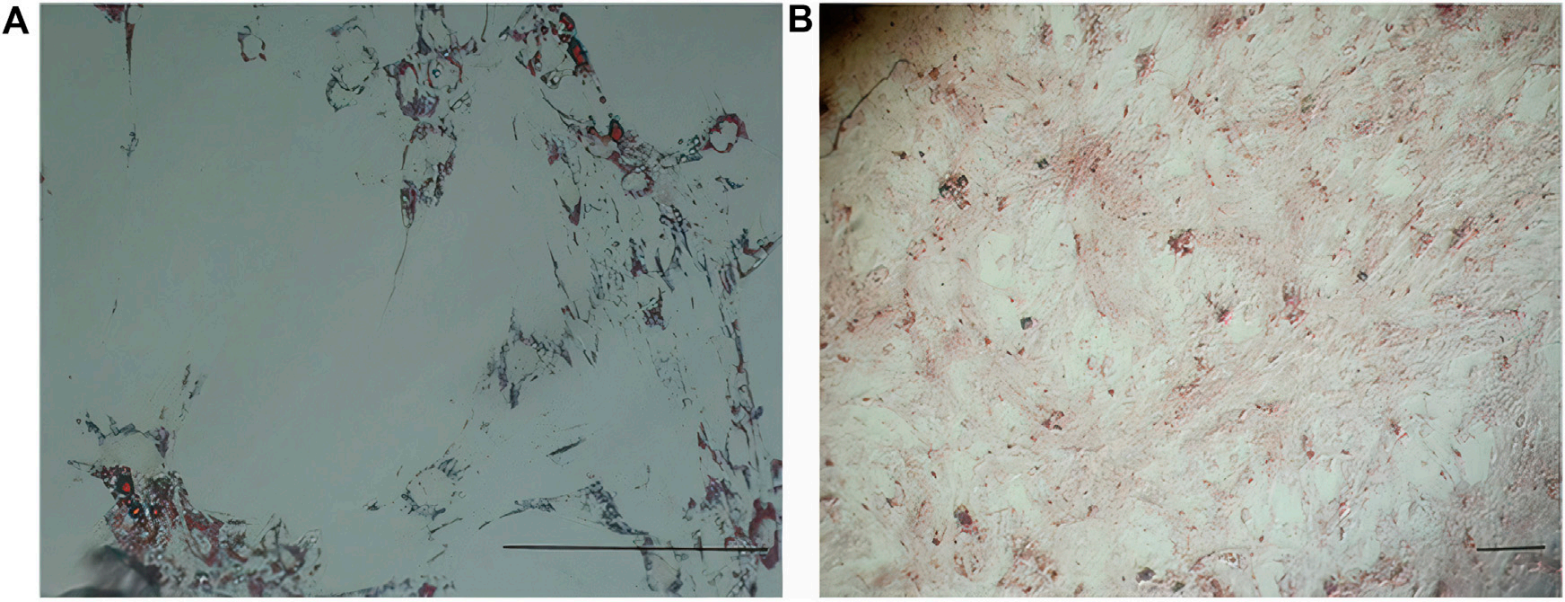
**(A)** Differentiation of MSCs into adipocytes (empty space with a thin rim of cytoplasm close to the basal lamina) revealed after Oil Red O staining. **(B)** Differentiation of MSCs into osteocytes (stellate or star-shaped cells with multiple slender or cytoplasmic processes projected in different directions) revealed after alizarin red staining. Scale bar shows 100 μm (magnification is 360x).

**FIGURE 3:**
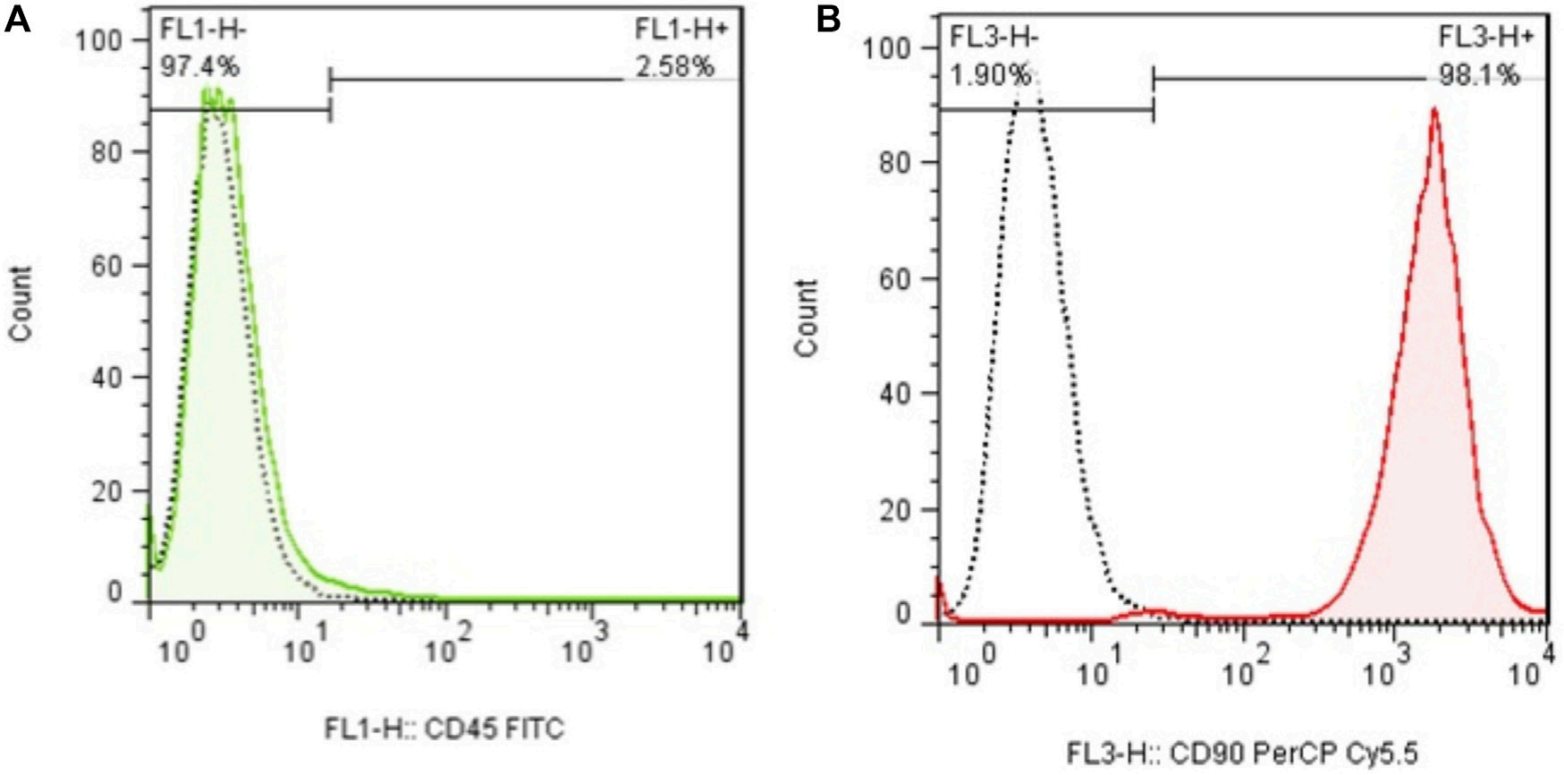
**(A)** Flow cytometry analysis of MSCs with CD45 and **(B)** CD90. The lack of expression of CD44, in addition to the expression of CD90 in these cells, shows the mesenchymal origin of these stem cells.

In early cultures, cells were adhered to the culture flask with different morphologies, including flattened, spindle, and multi-dimensional. In the following subcultures, the number of spindle-shaped or fibroblastic-like cells, which is the typical appearance of MSCs, was gradually increased, and eventually, this shape became the dominant appearance at P3 (Figure 1). Cells at P3 showed the capability of differentiation toward the adipogenic lineage. The first changes in the appearance of cells to adipocyte formation were seen only 3 days after exposure to the specific culture medium. In the following weeks, lipid droplets gently covered the flask surface. Most of the cells contained such droplets at the end of the third week. Staining of the cells with Oil Red O dye confirmed the existence of lipid droplets in newly formed adipocytes. Regarding osteogenic differentiation, the first signs of osteocyte formation were seen in the first week after treating cells with the specific medium. Aggregates of osteocytes emerged and expanded gradually, and ultimately, they were evident at the end of the third week. Alizarin red staining confirmed the existence of osteocytes (Figure 2). In order to finalize the characterization of MSCs, the expression of mesenchymal-specific markers was examined by flow cytometry. As shown in Figure 3, the lack of expression of CD45, along with the expression of CD90 by the proliferated cells at P3, showed their mesenchymal origin.

### MTT assay

**FIGURE 4:**
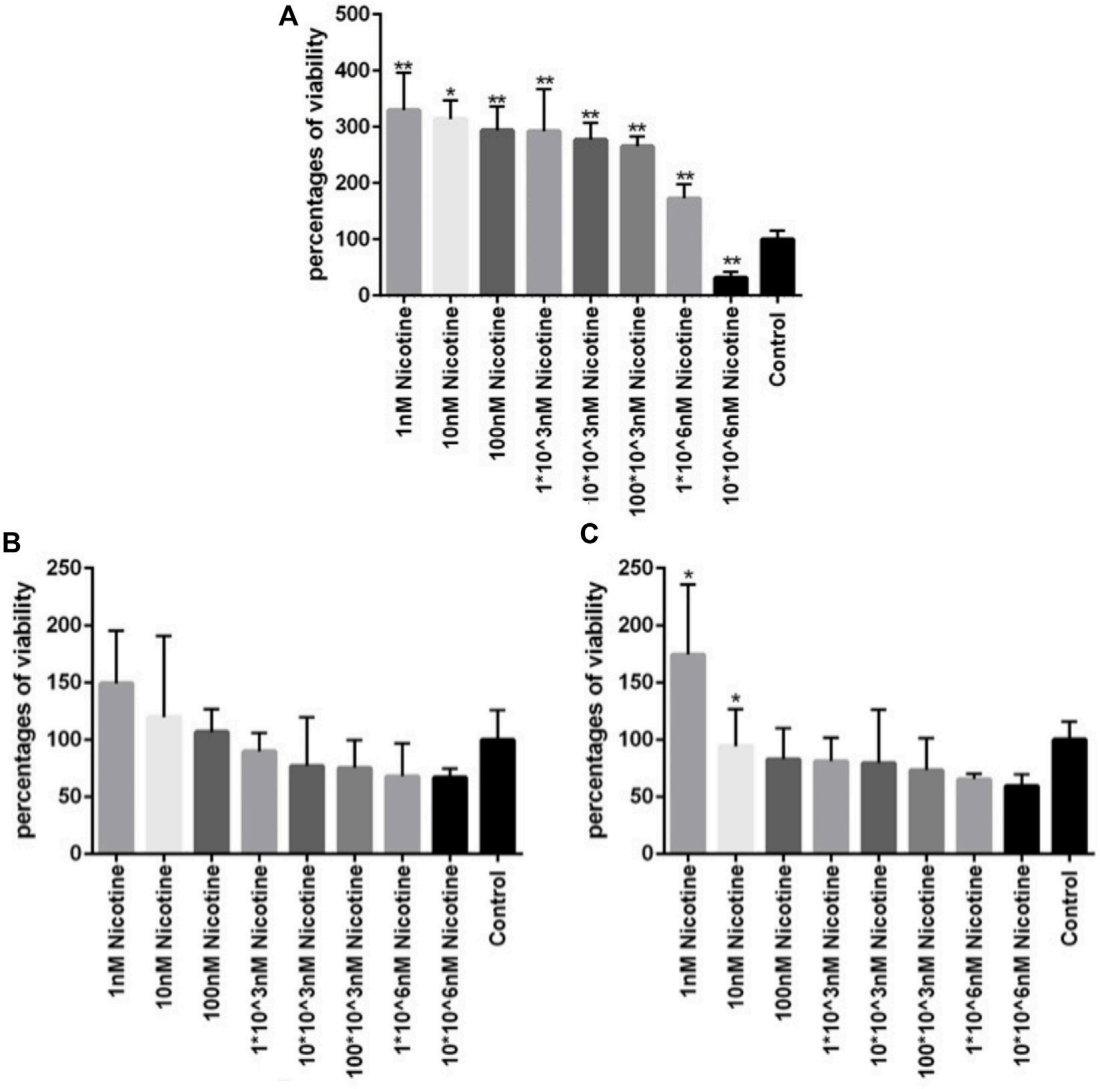
**(A, B, C)** Viability of MSCs after 1, 2, and 3 days of exposure, respectively, to different concentrations of nicotine. ANOVA was used for statistical analysis. Asterisks show statistical significance in comparison to the control group. One star symbol (*) indicates a *p*-value < 0.05, while two star symbols (**) represent a *p*-value < 0.005.

To investigate the viability of MSCs after being exposed to different concentrations of nicotine and find the concentration of interest for downstream experiments, an MTT assay was carried out. After MSC treatment with nicotine for 1, 2, and 3 days, viability was checked in the corresponding time frames. In all three days (Figure 4), the percentage of viability decreased with increasing nicotine concentrations from 1 nM to 10*10^6^ nM. Moreover, MSCs showed higher viability on day 1 compared with days 2 and 3 at similar nicotine concentrations. In other words, viability was highest in the first 24 h and then gradually decreased in the following days. Finally, a nicotine concentration of 100 nM was determined for upcoming experiments.

### Growth curve and population doubling time

**FIGURE 5:**
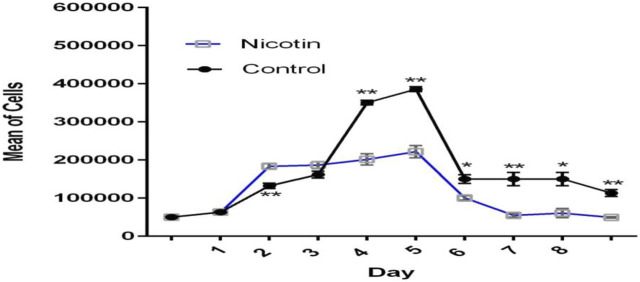
Growth curve of MSCs exposed to nicotine (100 nM). Although excitatory in the early stage, the inhibitory effect of nicotine on MSC growth was evident afterward.

MSCs were exposed to 100 nM nicotine in the case group, while the control group contained PBS instead. The growth curve showed that cells in the control group were in the lag phase on the first day and then started to grow until day 5. In the following days, the growth was reduced and reached the plateau phase. In general, the overall trend in the case group was similar to that of the control group but with a diminished cell number. The lag phase lasted for one day for this group. During the early exponential phase from days 1–2, cells in the case group outnumbered their control peers. Later, however, they became lower in number compared with the control group (Figure 5). PDT of the case group (164.76 h) was nearly double that of the control counterpart (81.16) after 8 days (Table 2).

**TABLE 2:** Population doubling time in the case and control groups.

Day	Case group (h)	Control group (h)
1	7.84	9.26
2	15.55	16.66
3	22.52	18.02
4	28.78	23.22
5	54.68	43.32
6	108.90	51.98
7	114.77	60.65
8	164.76	81.16

MSCs in the case group need more time (164.76 h) for population doubling than cells in the control group (81.16 h) at the end of day 8. (h: hours).

### Morphology of MSCs on days 3 and 7

**FIGURE 6:**
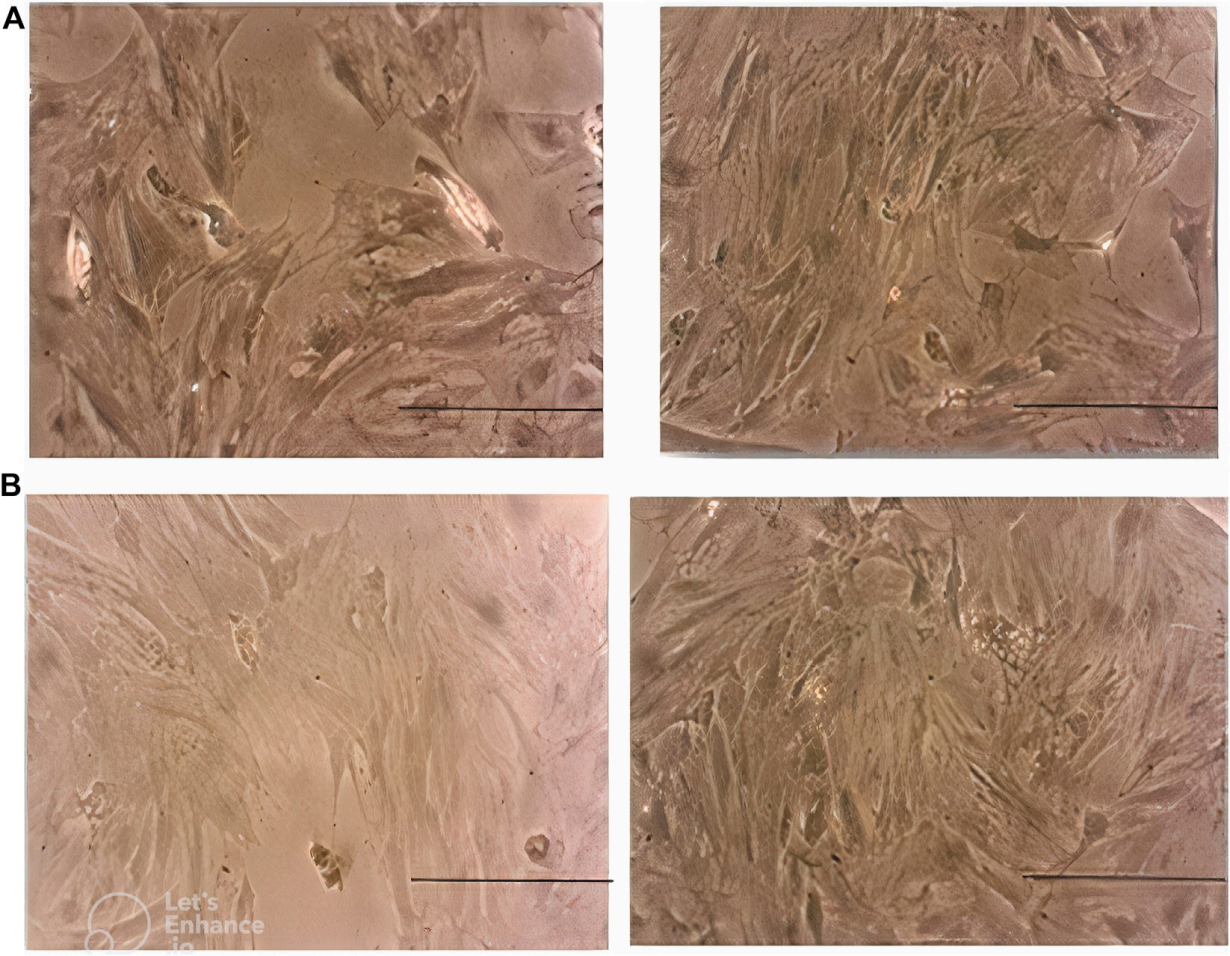
**(A)** Morphology of MSCs in the case group (left) and control group (right) on day 3. Note the lower density of cells in the case group. **(B)**: morphology of MSCs in the case group (left) and control group (right) on day 7. Note the lower density, paleness, and disruption of cells in the case group. Scale bar shows 100 μm (magnification is 360x).

Morphological assessment of MSCs on day 3 revealed lower cell density in the case group compared with the control peers. This difference became more prominent on day 7. Moreover, MSCs in the case group seemed to be pale and disrupted compared to the cells in the control group, especially on day 7 (Figure 6).

### Expression of cardiac-specific genes

**FIGURE 7:**
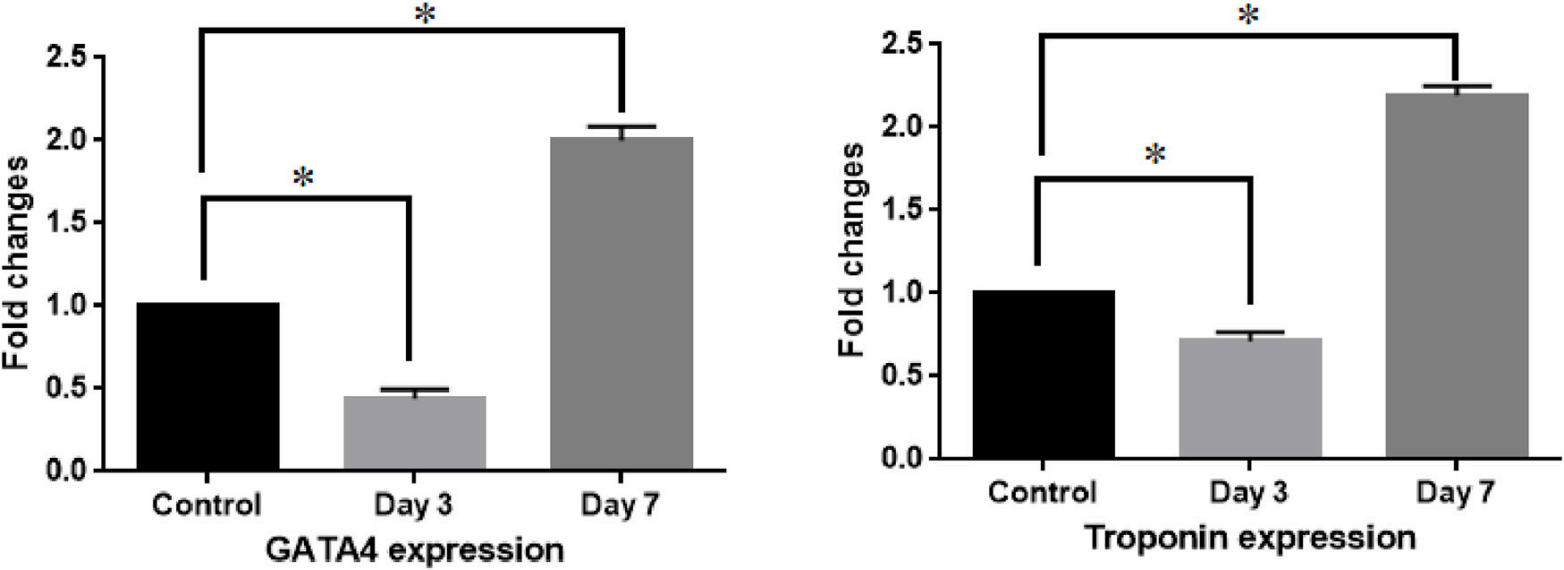
Differential expression of cardiac genes between the case and control groups on days 3 and 7. Fold changes were calculated via 2^−ΔΔCt^. Asterisk shows statistical significance compared with the control group. ANOVA was used for statistical comparison.

Following the induction of cardiac differentiation by adding 5-azacytidine in both the case and control groups, the expression of GATA4 and troponin as two successive markers in the differentiation process was examined. Analysis showed that GATA4 expression in the case group was nearly half (0.44) of that in the control group (statistical significance) on day 3, and this ratio reversed on day 7. It means that GATA4 expression was double (1.98) in the case group *versus* the control peers. Similarly, although troponin was expressed at a significantly lower level in the case group on day 3 (0.71) compared to the control group, this increased to 2.19-fold on day 7 as well (Figure 7).

## Discussion

Approximately 8.7 million deaths and 239 million DALYs were attributed to tobacco usage in 2019. CVD is the underlying reason for 36.7% of the attributed deaths. Tobacco increases the incidence of premature mortality. In total, the epidemic status of using tobacco, with one billion active smokers, has become a global health challenge, and unfortunately, it continues to escalate (Roth et al., 2020). Over 7,000 chemicals and approximately 250 known toxins are found in cigarette smoke (Wahl et al., 2016). Although a substantial amount of knowledge has been documented about the adverse effects of nicotine on health, there is a scarcity of data regarding the impact of this addictive substance on stem cells. The prominent properties of stem cells have opened a bright horizon for restoring lost functions after injury. This is particularly pronounced in organs with limited regenerative capacity, like the heart.

Despite enormous advancements in stem cell therapy for cardiac injuries during the last decade, there is still a long way toward clinical translation, possibly due to the existence of comorbidities and the style of living of recipients. As smokers constitute a common population among cardiovascular patients, it is vital to understand the barrier effect of nicotine on receiving the maximum efficiency of stem cell therapy. Because proliferation and differentiation are two fundamental elements in delivering the regenerative benefits of MSCs (Chamberlain et al., 2007), we aimed to investigate the effect of nicotine in a concentration near that of smokers’ blood on the viability and cardiac differentiation of MSCs.

Our findings show that nicotine inhibits the normal growth of MSCs. In line with this finding, it was also revealed that nicotine-treated MSCs need considerably more time for population doubling. Another study reported that MSCs isolated from the periodontal ligament of the oral cavity, which are in close contact with nicotine, have reduced regenerative potential (Benowitz et al., 2009). Studies demonstrated that a nicotine concentration of 0.5–1.5 mg/mL (3–9 mM) decreased cell proliferation and increased apoptosis (Zeng et al., 2014). Decreased proliferation was significant even at nicotine concentrations as low as 0.1–10 mM (Kim et al., 2013). In addition, short-time (24 h) exposure of MSCs to nicotine impairs proliferation and induces apoptosis in MSCs (Schraufstatter et al., 2010). Such diminished proliferation was evident in MSCs derived from smokers compared with cells isolated from nonsmokers. Interestingly, the effect of nicotine on the proliferation capacity of MSCs seems to be long-lasting. It was found that MSCs did not regain the normal proliferation condition after more than 300 times of sub-culturing (Ng et al., 2015).

On the contrary, exposing MSCs to 50–100 nM nicotine for a period of 7 days significantly increased the cell number (Shen et al., 2013). In our study, 100 nM of nicotine increased proliferation in the early days of exposure. However, a decreasing trend in proliferation was seen afterward. Another study reported that a concentration of 1–100 μM did not alter proliferation, while doses of 1–2 mM had an increased effect, and there was a decreased proliferation in concentrations of over 5 mM (Kim et al., 2012). In summary, it seems that there is a certain threshold for cigarette smoke to be toxic for the MSC population (Wahl et al., 2016). Such a variety of findings should be partly sought in different species and tissue sources of cells among studies. In addition, decreased proliferation after nicotine exposure may be explained by the generation of reactive oxygen species (Shen et al., 2013) or changes in the nicotine-induced cell cycle (Zeng et al., 2014). Cell morphology (cytoplasmic vacuole formation and pyknotic nuclei) was also altered upon exposure to nicotine. These impairments may be related to the deregulated expression of certain markers involved in cell structure and movement (Zeng et al., 2014).

It was also demonstrated that nicotine impairs the *in vitro* differentiation of MSCs. Differentiation of MSCs to chondrogenic lineages was hampered in a concentration-dependent manner (Deng et al., 2012; Kim et al., 2013). Cigarette smoking also undermines the chondrogenic differentiation ability of MSCs (Chamberlain et al., 2007). This attenuated capability, which represents the reduced generation of matrix proteoglycan or collagen, should be considered in the inefficient treatment strategies of diseases like osteoarthritis using stem-cell-based approaches (Greenberg et al., 2017). Notably, studies revealed that smoking seriously deteriorates bone health via modulating normal reparative bone formation, and this may be the underlying reason for the predisposition of smokers to osteoporosis (Ayo-Yusuf and BJNJoCP, 2014) and delayed healing following fractures (Pearson et al., 2016). The reasons for such delays should be sought in the inability of MSCs to efficiently differentiate toward the osteogenic lineage (Ng et al., 2015; Wahl et al., 2016). Smoker-derived MSCs generated significantly less bone matrix in comparison to cells isolated from nonsmokers (Zhao et al., 2018). Upon autologous implantation of these scaffolds, the stability of implants was higher in nonsmokers (Chan and Huang, 2020). Intriguingly, smoker-derived MSCs produced an elevated level of lipid contents even after several weeks of culture in a nicotine-free medium compared with cells isolated from nonsmokers (Ng et al., 2015).

To the best of our knowledge, there are no reports regarding the impact of nicotine on the cardiogenic differentiation of MSCs. In our study, the expressions of two cardiac-specific markers were examined. GATA4, which was expressed at the early stages of cardiac differentiation, was significantly downregulated on day 3 in MSCs. This was the case for troponin as well, which is a late cardiac marker. In total, the findings revealed the inhibitory effect of nicotine on cardiac differentiation in MSCs. However, increased expression of both GATA4 and troponin in the case group on day 7 may be explained by the decreasing effect of nicotine after 7 days. It seems that, similar to proliferation, there is a threshold of nicotine concentrations for affecting the potential of MSC differentiation. The capacity of adipogenic differentiation was not significantly altered after 21 days of exposure to 0.5% cigarette smoking extract (Wahl et al., 2016).

Nicotine mediates its biological effects through nicotine acetylcholine receptors (nAChRs) (Albuquerque et al., 2009), and stem cells such as MSCs express the subunits of these receptors (Hoogduijn et al., 2009; Carballosa et al., 2016). nAChRs provide a suitable bridge for connecting nicotine to the stem cells. Although these receptors are specifically designed for Ca21 ions, nicotine acts as an agonist, resulting in the elevation of intracellular calcium upon activation. The upcoming alteration in intracellular pathways leads to increased secondary messenger cAMP and phosphorylation of ERKs (Hoogduijn et al., 2009). Thus, ERKs are substantially involved in stem cell proliferation and differentiation (Michailovici et al., 2014).

Many unknown factors have been elucidated in the area of efficient stem-cell-based therapies, but there is much more that remains to be discovered. Some of these issues include the translation of *in vitro* findings into *in vivo* settings and assessing the effects of nicotine in acute *versus* chronic conditions. Current data demonstrate the adverse effects of nicotine on the regenerative potential of stem cells, a composite of proliferation, migration, and differentiation abilities. On a clinical scale, patients who are candidates for stem cell therapies may have been smokers for a prolonged time. This chronic exposure to nicotine should be considered at the time of autologous stem cell therapy. Further investigations may help develop stem cell banks for allogenic transplantation and, ultimately, optimize stem cell therapy for cardiovascular patients.

## Conclusion

Nicotine imposes an inhibitory effect on the normal proliferation and cardiac differentiation of MSCs. This aspect is of prominent value to smokers who are candidates for stem cell therapy because of CVD.

## Data Availability

The original contributions presented in the study are included in the article/Supplementary Material; further inquiries can be directed to the corresponding author.

## References

[B1] Albuquerque E. X. Pereira E. F. Alkondon M. Rogers S. W. J. P. R. (2009). Mammalian nicotinic acetylcholine receptors: from structure to function. Physiol. Rev. 89 (1), 73–120. 10.1152/physrev.00015.2008 19126755 PMC2713585

[B2] Ayo-Yusuf O. BjnjoCP O. (2014). Epidemiological association between osteoporosis and combined smoking and use of snuff among South African women. Niger. J. Clin. Pract. 17 (2), 174–177. 10.4103/1119-3077.127542 24553027

[B3] Benowitz N. L. Hukkanen J. Jacob P. J. N. P. (2009). Nicotine chemistry, metabolism, kinetics and biomarkers. Handb. Exp. Pharmacol. 192, 29–60. 10.1007/978-3-540-69248-5_2 PMC2953858 19184645

[B4] Carballosa C. M. Greenberg J. M. Cheung HSJAb (2016). Expression and function of nicotinic acetylcholine receptors in stem cells. AIMS Bioeng. 3 (3), 245–263. 10.3934/bioeng.2016.3.245

[B5] Chamberlain G. Fox J. Ashton B. Middleton J. J. S. C. (2007). Concise review: mesenchymal stem cells: their phenotype, differentiation capacity, immunological features, and potential for homing. Stem Cells 25 (11), 2739–2749. 10.1634/stemcells.2007-0197 17656645

[B6] Chan A. H. Huang NFJRm (2020). Effects of nicotine on the translation of stem cell therapy. Regen. Med. 15 (5), 1679–1688. 10.2217/rme-2020-0032 32618492 PMC7466930

[B7] Deng Y. Li T.-Q. Yan Y.-E. Magdalou J. Wang H. Chen L.-B. J. B.-M. M. (2012). Effect of nicotine on chondrogenic differentiation of rat bone marrow mesenchymal stem cells in alginate bead culture. Biomed. Mat. Eng. 22 (1-3), 81–87. 10.3233/BME-2012-0692 22766705

[B8] Fagerström K. O. Hughes JRJN Research T. (2002). Nicotine concentrations with concurrent use of cigarettes and nicotine replacement: a review. Nicotine Tob. Res. 4 (2), S73–S79. 10.1080/1462220021000032753 12573169

[B9] Fu X. Zong T. Yang P. Li L. Wang S. Wang Z. (2021). Nicotine: regulatory roles and mechanisms in atherosclerosis progression. Food Chem. Toxicol. 151, 112154. 10.1016/j.fct.2021.112154 33774093

[B10] Greenberg J. M. Carballosa C. M. Cheung HSJSctm (2017). Concise review: the deleterious effects of cigarette smoking and nicotine usage and mesenchymal stem cell function and implications for cell-based therapies. Stem Cells Transl. Med. 6 (9), 1815–1821. 10.1002/sctm.17-0060 28696009 PMC5689746

[B11] Hoogduijn M. J. Cheng A. Genever P. G. J. S. C. (2009). Functional nicotinic and muscarinic receptors on mesenchymal stem cells. Stem Cells Dev. 18 (1), 103–112. 10.1089/scd.2008.0032 18393628

[B12] Kim B.-S. Kim S.-J. Kim H.-J. Lee S.-J. Park Y.-J. Lee J. (2012). Effects of nicotine on proliferation and osteoblast differentiation in human alveolar bone marrow-derived mesenchymal stem cells. Life Sci. 90 (3-4), 109–115. 10.1016/j.lfs.2011.10.019 22115820

[B13] Kim D. H. Liu J. Bhat S. Benedict G. Lecka-Czernik B. Peterson S. J. (2013). Peroxisome proliferator-activated receptor delta agonist attenuates nicotine suppression effect on human mesenchymal stem cell-derived osteogenesis and involves increased expression of heme oxygenase-1. J. Bone Min. Metab. 31, 44–52. 10.1007/s00774-012-0382-0 22945906

[B14] Lee J. Cooke JPJA (2011). The role of nicotine in the pathogenesis of atherosclerosis. Atherosclerosis 215 (2), 281–283. 10.1016/j.atherosclerosis.2011.01.003 21345436 PMC3755365

[B15] Madrigal M. Rao K. S. Nhjjotm R. (2014). A review of therapeutic effects of mesenchymal stem cell secretions and induction of secretory modification by different culture methods. J. Transl. Med. 12 (1), 1–14. 10.1186/s12967-014-0260-8 25304688 PMC4197270

[B16] Michailovici I. Harrington H. A. Azogui H. H. Yahalom-Ronen Y. Plotnikov A. Ching S. (2014). Nuclear to cytoplasmic shuttling of ERK promotes differentiation of muscle stem/progenitor cells. Development 141 (13), 2611–2620. 10.1242/dev.107078 24924195 PMC4067960

[B17] Ng T. K. Huang L. Cao D. Yip Y. W.-Y. Tsang W. M. Yam G. H.-F. (2015). Cigarette smoking hinders human periodontal ligament-derived stem cell proliferation, migration and differentiation potentials. Sci. Rep. 5 (1), 7828–7837. 10.1038/srep07828 25591783 PMC5379007

[B18] Onor I. O. Stirling D. L. Williams S. R. Bediako D. Borghol A. Harris M. B. (2017). Clinical effects of cigarette smoking: epidemiologic impact and review of pharmacotherapy options. Int. J. Environ. Res. Public Health 14 (10), 1147. 10.3390/ijerph14101147 28956852 PMC5664648

[B19] Pearson R. G. Clement R. Edwards K. Bejbo S. (2016). Do smokers have greater risk of delayed and non-union after fracture, osteotomy and arthrodesis? A Syst. Rev. meta-analysis 6 (11), e010303. 10.1136/bmjopen-2015-010303 PMC5129177 28186922

[B20] Roth G. A. Mensah G. A. Johnson C. O. Addolorato G. Ammirati E. Baddour L. M. (2020). Global burden of cardiovascular diseases and risk factors, 1990–2019: update from the GBD 2019 study. J. Am. Coll. Cardiol. 76 (25), 2982–3021. 10.1016/j.jacc.2020.11.010 33309175 PMC7755038

[B21] Santanam N. Thornhill B. A. Lau J. K. Crabtree C. M. Cook C. R. Brown K. C. (2012). Nicotinic acetylcholine receptor signaling in atherogenesis. Atherosclerosis 225 (2), 264–273. 10.1016/j.atherosclerosis.2012.07.041 22929083

[B22] Schraufstatter I. U. DiScipio R. G. Skjjosc K. (2010). Alpha 7 subunit of nAChR regulates migration of human mesenchymal stem cells. J. Stem Cells 4 (4), 203–215. PMC2925301 20720594

[B23] Sensebé L. Gadelorge M. Fleury-Cappellesso S. J. S. therapy (2013). Production of mesenchymal stromal/stem cells according to good manufacturing practices: a review. Stem Cell. Res. Ther. 4 (3), 66–6. 10.1186/scrt217 23751270 PMC3707032

[B24] Shen Y. Liu H. X. Ying X. Z. Yang S. Z. Nie P. F. Cheng S. W. (2013). Dose‐dependent effects of nicotine on proliferation and differentiation of human bone marrow stromal cells and the antagonistic action of vitamin C. J. Cell. Biochem. 114 (8), 1720–1728. 10.1002/jcb.24512 23386463

[B25] Wahl E. A. Schenck T. L. Machens H.-G. Egaña J. T. J. S. R. (2016). Acute stimulation of mesenchymal stem cells with cigarette smoke extract affects their migration, differentiation and paracrine potential. Sci. Rep. 6 (1), 22957. 10.1038/srep22957 26976359 PMC4791635

[B26] Whitehead A. K. Erwin A. P. Yue XJAP (2021). Nicotine and vascular dysfunction. Acta Physiol. (Oxf). 231 (4), e13631. 10.1111/apha.13631 33595878 PMC8026694

[B27] Zeng H. L. Qin Y. L. Chen H. Z. Bu Q. Q. Li Y. Zhong Q. (2014). Effects of nicotine on proliferation and survival in human umbilical cord mesenchymal stem cells. J. Biochem. Mol. Toxicol. 28 (4), 181–189. 10.1002/jbt.21551 24488958

[B28] Zhao X. Zhu B. Duan Y. Wang X. Djbri L. (2018). The effect of smoking behavior on alveolar bone marrow mesenchymal stem cells of clinical implant patient. Biomed. Res. Int. 2018, 7672695. 10.1155/2018/7672695 30584539 PMC6280244

